# Type A aortic dissection during diagnostic coronary angiography in normal coronary arteries: A case report

**DOI:** 10.1002/ccr3.7777

**Published:** 2023-08-06

**Authors:** Alena Gonzalez, Xiongbin Lin, Vikash Jaiswal, Abhigan Babu Shrestha, Yeonah Song, David Song

**Affiliations:** ^1^ Department of Internal Medicine Icahn School of Medicine at Mount Sinai – Elmhurst Hospital Center Elmhurst New York USA; ^2^ Department of Cardiovascular Research Larkin Community Hospital South Miami FL USA; ^3^ Department of Internal Medicine M Abdur Rahim Medical College Dinajpur Bangladesh; ^4^ St John's University Queens New York USA

**Keywords:** aortic dissection, coronary angiography, iatrogenic

## Abstract

**Key Clinical Message:**

Aortic dissection is one of the rare complication of coronary angiography. The main treatment underlies surgical intervention however; management should be done with patient's decision.

**Abstract:**

Coronary angiography (CA) is a widely utilized diagnostic procedure used to evaluate blood flow through the coronary arteries and detect coronary artery disease (CAD). Despite its widespread use, it has complications including non‐life‐threatening, while there are some rare complications that can occur. We present a case of an elderly woman who presented with ST elevation myocardial infarction (STEMI) and underwent CA without evidence of CAD. However, the patient later developed type A aortic dissection (AD) as a complication of the procedure. Surgery is considered emergent treatment for type A‐AD, but the patient was asymptomatic without any complaint, shared decision making was conducted and the patient decided to pursue conservative treatment without surgical intervention. Therefore, managing AD should be tailored to each patient individually through shared‐decision making.

## Graphical abstract

Figure (A, B) CT angio of abdomen and pelvis with contrast showing type A aortic dissection.

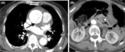

## 1 INTRODUCTION

Coronary artery disease (CAD) is a significant public health problem worldwide, ranking as the third leading cause of death globally. The gold standard for diagnosis of CAD is coronary angiography (CA), a non‐invasive diagnostic procedure that uses contrast dye and radiographic technology to evaluate blood flow through the coronary arteries.^1^ Complications are usually non‐life threating such as soreness at the site of catheterization, mild kidney injury, minimal risk of bleeding, and injury to the artery. There are some rare complications that can occur including isolated aortic dissection (AD), which has been reported in the medical literature as a rare but serious complication of CA.

Aortic dissection is defined as a disruption or injury to the intima layer of the aorta, resulting in bleeding within the wall of the vessel and the formation of a false lumen. Diagnosis of AD can be made using a variety of modalities, including transesophageal echocardiogram, computerized tomography (CT) scan of the chest, and magnetic resonance angiogram (MRA), which is considered the gold standard with a sensitivity and specificity of 98%.^2^ AD can be classified using the Stanford and DeBakey systems. According to the Stanford classification, there are two types of AD: Type A which involves the aortic arch and thoracic‐abdominal aorta, and type B which only involves the abdominal aorta distal to the left subclavian artery and does not involve the aortic arch. Further subclassification using DeBakey's includes three types: Type I, involving the aortic arch and thoracoabdominal aorta and may progress to involve the abdominal aorta; Type II, involving only the abdominal aorta; Type IIIa, involving the descending aorta, proximal to the celiac artery and distal to the left subclavian artery and Type IIIb, involving the thoracoabdominal aorta, distal to the celiac artery.^3^ The potential causes of AD include genetic disorders, hypertension, and trauma or iatrogenic causes. Isolated AD is a rare complication of CA, with the potential for fatal outcomes if not recognized promptly. Treatment options for AD vary depending on the type, location, and severity of the dissection. Conventionally, surgical intervention is typically reserved for type A dissections, while type B dissections are often managed medically, and surgical intervention may not be necessary.

We present a case of an 89‐year‐old woman complaining of generalized weakness associated with left arm pain and shortness of breath. Electrocardiogram (ECG) changes concerning myocardial ischemia were noted, and the patient underwent CA without complications and evidence of CAD. A post‐procedure transthoracic echocardiogram (TTE) revealed isolated type A‐AD without coronary artery involvement. This case highlights the importance of continued monitoring of patients following CA to surveillance and manage any potential complications, including the rare but serious complication of AD. Further research is needed to better understand the incidence, risk factors, and management of this complication.

## 2 CASE PRESENTATION

**FIGURE 1:**
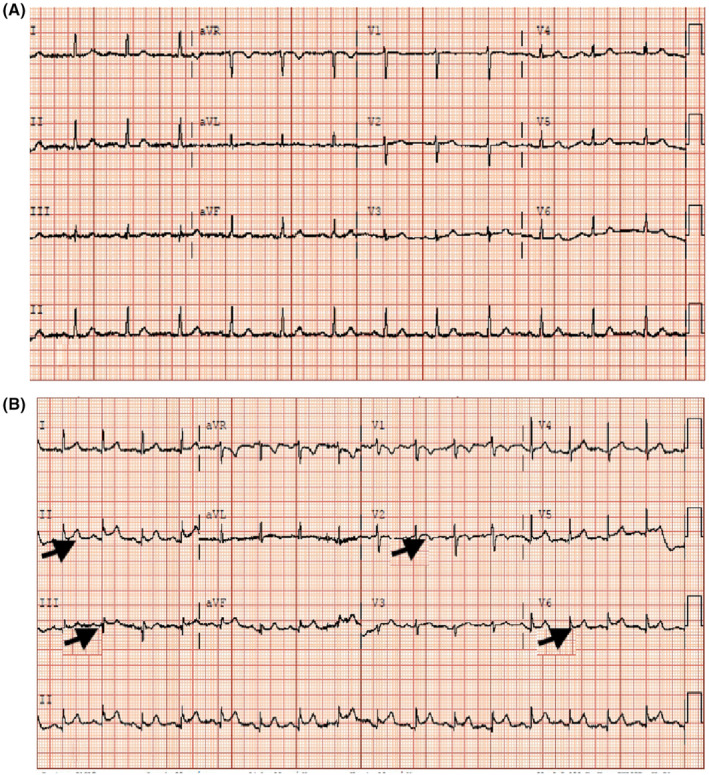
Electrocardiogram (ECG) showing (A) Sinus Rhythm without ST elevation (B) ST elevation in leads II, III, and V6, as well as T wave inversions in lead V2.

**FIGURE 2:**
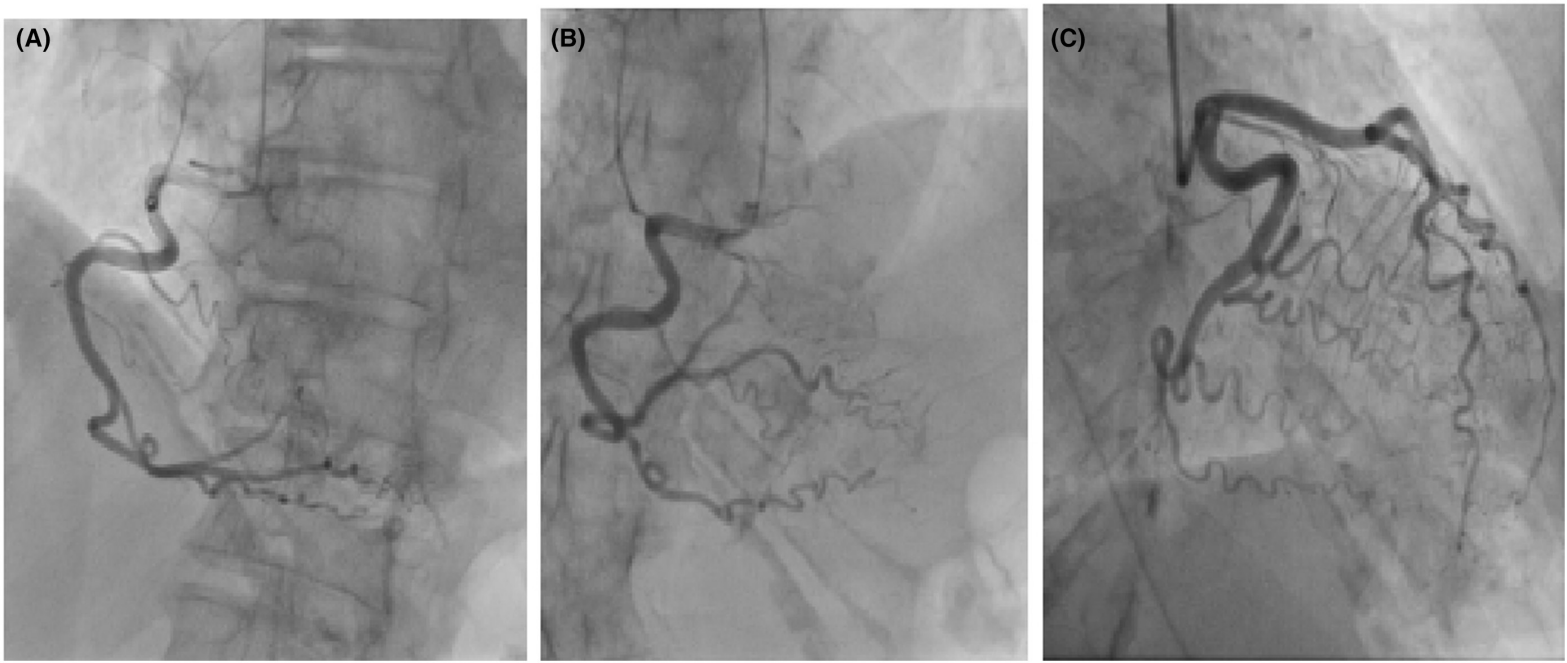
Coronary angiography showing patent coronaries arteries without signs of obstruction (A) patent right coronary artery (RCA), and (B) posterior circumflex (C) patent left anterior descending (LAD).

An 89‐year‐old woman with a history of asthma and hypertension presented with complaints of decreased oral intake, left arm pain, chest tightness, and shortness of breath for 3 days. Vital signs on admission were unremarkable (blood pressure: 128/75 mmHg, heart rate: 96 bpm, temperature: 98.6 F, oxygen saturation: 98%). Laboratory results revealed serum creatinine of 0.90 mg/dL, uptrending troponin levels of 0.021 ng/mL to 0.023 ng/mL and Pro‐BNP (1990 pg/mL). Initial ECG revealed normal sinus rhythm without signs of ischemia (Figure 1A). However, repeated ECG revealed sinus tachycardia at a rate of 100 beats per minute with ST elevation in leads II, III, and V6, as well as T wave inversions in lead V2 (Figure 1B). STEMI code was activated, cardiology was consulted, and subsequently admitted to the cardiac care unit and urgently taken to cardiac cath lab. In interim, aspirin 325 mg orally and heparin 4000 units were administered as an intravenous (IV) bolus. The patient then underwent an emergent CA via a left radial artery, which revealed patent, normal coronaries arteries (Figure 2A–C) with left ventricular ejection fraction of 55%, and no aortic stenosis or wall motion abnormalities. The patient tolerated the procedure well, with no complications reported. Post‐CA labs and vitals were unremarkable and TTE was recommended in addition to aggressive CAD risk modifications and medical therapy.

**FIGURE 3:**
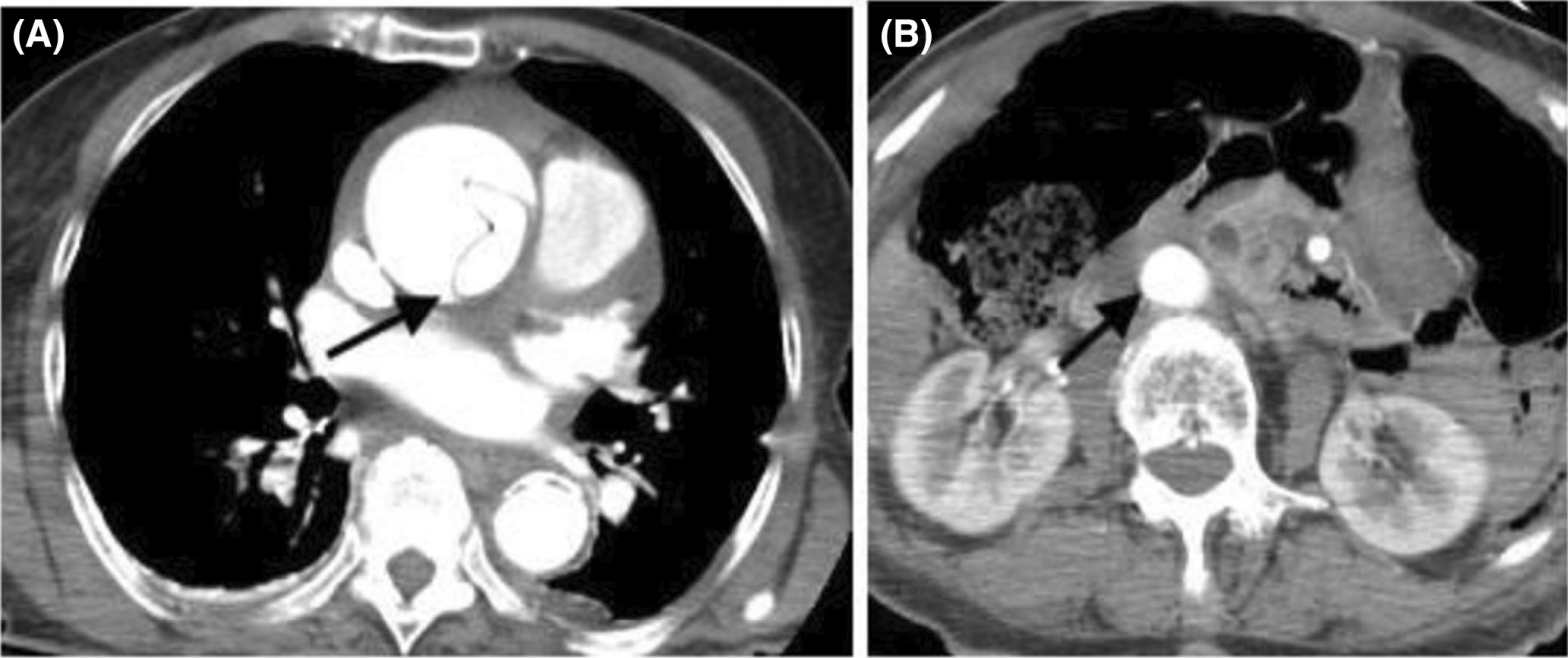
(A, B) CT angio of abdomen and pelvis with contrast showing type A aortic dissection.

Transthoracic echocardiogram revealed a dissection flap located in the ascending aorta, extending to the superior aspect of the aortic root, consistent with Stanford Type A‐AD. Cardiothoracic surgery was urgently consulted for surgical treatment options and recommended a computerized tomography angiography (CTA) of the chest, abdomen and pelvis with contrast to further evaluate the dissection. The CTA partially revealed a Stanford Type A‐AD at the aortic root level, with fluid and heterogeneous material surrounding the image portion of the affected aorta, while the abdominal aorta was intact (Figure 3B).

Given the patient's hemodynamic stability and absence of symptoms, a multidisciplinary team approach was taken in order to discuss the goals of care with the patient and family members. Considering the patient's advanced age and comorbidities; she was deemed as a high risk surgical candidate and surgical management was deferred. Instead, conservative medical management was offered as an alternative. Patient remained stable, she was made do not resuscitate/do not intubate (DNR/DNI) and was discharged to a subacute rehabilitation center on metoprolol tartrate 25 mg PO daily, atorvastatin 40 mg daily, aspirin 81 mg daily, and amlodipine 5 mg daily. Patient was discharged with Cardiology follow up and is doing well. Unfortunately, the patient was lost to follow up without any further evidence of hospitalization.

## 3 DISCUSSION

We came across multiple medical literature and case reports on this topic. However, in most cases reported, patients had either coronary or subclavian artery involvement, a history of CAD, or symptoms/signs were noticed amid the procedure.

Aortic dissection is a rare complication of CA with an extremely low incidence rate. According to the International Registry of Acute Aortic Dissection (IRAD), which collected data from 1996 to 2016, reported that only 2.5% of acute AD cases were attributed to iatrogenic causes, with 75% of cases being classified as Type A dissections.^4^ In addition, it was found that the incidence of AD following diagnostic procedures, such as CA, is lower compared to interventional procedures like PCI which occurs at a rate of 0.12% compared to a lower rate after diagnostic procedures at only 0.01%.^4^, ^5^

The exact mechanism and etiology for the development of AD following diagnostic procedures remains unclear, but potential contributing factors include aging‐related atherosclerosis changes and catheter‐induced trauma. It has been established that advanced age is a major risk factor for atherosclerotic cardiovascular changes; it is hypothesized that this relationship results from feedback dysregulation between L‐6 and vascular mitochondrial union resulting in atherogenesis, which is affected by aging.^6^ On the other hand, using non‐conventional catheters has been associated with a higher incidence of AD. Although our patient did not have evidence of obstructive CAD, it is safe to assume that given the patient's age and comorbidities, the likelihood of atherosclerotic cardiovascular changes is high thus increasing risk for artery trauma/perforation during diagnostic CA.

It is important to note that the treatment and management of AD varies greatly depending on the extent and involvement of the dissection. Type‐A AD carries a mortality rate of around 50%, thus rapid evaluation with imaging studies and surgical consult are needed immediately.^3^ However, certain clinical scenarios may warrant a different approach to management. In our case, given the patient's age, hemodynamic stability, and absence of symptoms, shared decision‐making was conducted to assess the risk and benefit of surgical intervention and ultimately the patient decided on a conservative management. Patient's vital signs prior to discharge were stable with the goal to maintain the systolic blood pressure in 120–130 and heart rate in 70–80. Unfortunately, given the lack of outpatient follow up, we were unable to monitor her symptoms. However, there were no further reported hospitalizations which ensured that the patient was stable. This case alludes to risk stratifying patients based on symptoms and patient's decision instead of radiographic diagnosis alone. Even patients without evidence of coronary artery disease can have a risk to develop type A‐AD. In cases of asymptomatic radiographic diagnosis of AD, shared decision‐making becomes even more important because it allows patients to be active participants in their healthcare and to make educated judgments about their care based on their values, preferences, and goals. In this case, the patient and family members were involved in the decision‐making regarding the treatment options for the AD. They were informed about the risks and benefits of surgical and medical management and were able to make an informed decision about the goal of care. Our case represents a complex scenario with multidisciplinary collaboration which resulted in a treatment plan that aligned with the patient's values, preferences, and goals and helped to ensure the best outcome for the patient.

## 4 CONCLUSION

Aortic dissection is a rare iatrogenic complication from CA and this case serves as a reminder of the importance of thorough diagnostic evaluation and the need for a multidisciplinary approach in managing complex cardiac cases using shared‐decision making.

## AUTHOR CONTRIBUTIONS

**Alena Gonzalez:** Writing – original draft; writing – review and editing. **Xiongbin Lin:** Writing – original draft; writing – review and editing. **Vikash Jaiswal:** Visualization; writing – original draft. **Abhigan Babu Shrestha:** Visualization; writing – review and editing. **Yeonah Song:** Writing – original draft; writing – review and editing. **David Song:** Writing – original draft; writing – review and editing.

## FUNDING INFORMATION

None.

## CONFLICT OF INTEREST STATEMENT

None.

## CONSENT

Written informed consent was obtained from the patient to publish this report in accordance with the journal's patient consent policy.

## Data Availability

Data is available on request from the corresponding author.
